# Association of 5-item Brief Symptom Rating Scale scores and health status ratings with burnout among healthcare workers

**DOI:** 10.1038/s41598-022-11326-1

**Published:** 2022-05-03

**Authors:** Meng-Ting Tsou

**Affiliations:** 1 Department of Family Medicine, MacKay Memorial Hospital, Taipei, Taiwan; 2 Department of Occupation Medicine, Mackay Medical College, Taipei, Taiwan; 3 Department of MacKay Junior College of Medicine, Nursing, and Management, New Taipei, Taiwan

**Keywords:** Psychology, Health care, Health occupations

## Abstract

This cross-sectional study evaluated and quantified the possible association of psychological symptoms and health status ratings on the burnout of healthcare workers from a tertiary medical center. Demographic data were obtained through a questionnaire survey. We evaluated their psychological symptoms using a 5-item Brief Symptom Rating Scale (BSRS-5) and burnout was measured using the Chinese version of the Maslach Burnout Inventory–Health Services Survey. The study began in Nov. 2018 and ended in Nov. 2020. A total of 2813 participants (men = 296, 10.5%; women = 2517, 89.5%) completed the questionnaires between December 2018 and March 2019. The response rate and complete rate were 71.7% and 93.2%. The multivariate analysis showed that, as the BSRS-5 level added, the odds ratio (OR) of burnout increased (BSRS-5 scores 6–9, 10–14, and 15–20; OR = 1.83, 3.23, and 9.15, separately; *p* value < 0.05). Overall, men (≥ 30 years of age) and women staffs with BSRS-5 scores ≥ 6; women with longer working hours (more than 46 h/week), men and women (≥ 30 years of age) working night shifts, and poor health status ratings were highly associated with burnout. The findings highlight the importance of screening for the BSRS-5 scores and health status ratings level for healthcare professionals at high risk of burnout, especially men ≥ 30 years of age and women with stressful working conditions.

## Introduction

Healthcare workers (HCWs) face a variety of psychosocial work hazards, including longer working hours, heavy workload, and shift work, which have been shown to increase burnout and other health risks than general workers^1–3^. Burnout is defined as a psychological syndrome derived from prolonged exposure to chronic stressors at work, which is evaluated by Maslach Burnout Inventory-Human Services Survey (MBI-HSS) and is often observed in healthcare workers (HCWs)^1,2,4,5^. Percentages of physicians and nurses working in intensive care units (ICU) with burnout according to the Stress and Burnout in Asian ICUs Study: China: 61.2; Hong Kong: 61.5; Japan: 41.2, Taiwan: 63.5. India: 55.4; Bangladesh: 34.6^1,2^. Around 79% of Taiwanese nurses reported above moderate burnout (including 19 medical centers, 77 regional hospitals, and 387 district hospitals)^4^. Personal burnout is around 40.3–57.9% among Taiwanese doctors from systemic review^5^.

In epidemiologic studies concerning the health risks of psychosocial work hazards, 5-item Brief Symptom Rating Scale (BSRS-5), health status ratings and burnout are commonly used outcome indicators in HCWs health surveys, but they capture different dimensions of subjective health^6–10^. The measurements of the BSRS-5, health status ratings, and burnout have been useful tools for identifying employees at high risk of sickness absence, cardiovascular diseases, musculoskeletal disorders, and other physical and mental ailments^6–10^.

BSRS-5 is often used in clinical settings to assess these symptoms, including insomnia, depression, hostility, anxiety, and interpersonal sensitivity; it is also used in mental health assessment interviews among HCWs^3,6,11,12^. The health status ratings are a composite indicator of the dimensions of physical and mental health^13,14^. Sleep-related issues are also strongly associated with physical and mental health^13^ and have been discussed separately in the contexts of quality of life, BSRS-5 and burnout^4,11,13^.

A cross-sectional study in Taiwan showed nurses with fair or poor self-rating health (SRH) was associated with an increased risk of BSRS-5 score ≧ 6 [odds ratio (OR) = 3.67; *p* < 0.001]^11^. Another study for Taiwanese employees showed that although SRH and burnout are highly correlated, the age-specific distribution patterns are quite different. Poor SRH increases with age and is associated with the presence of multiple disease symptoms. In contrast, a non-linear relationship was found between burnout and age, with employees aged 30–40 having higher burnout than other age group^13^. A systematic review and meta-analysis study have revealed that depression and anxiety could cause burnout among HCWs and different types of workers (such as teachers or firefighters)^15,16^. Thus, we suspected that psychological symptoms or severe physical diseases might also affect different burnout domains that are not directly related to emotional changes. This hypothesis should not be tested among participants with mental or uncontrolled physical illnesses, which might confound the results^6^.

There are significant gender-related differences between work and non-work stressors. Baruch et al. mentioned that women must take on more roles and face pressure from family and work simultaneously, which will lead to the poor mental health of women^17^. Traditionally, compared with the low demand and high control of men, the environment of high demand and low control of women is one of the reasons why women are more stressed than men^18^. Previous studies have investigated the potential moderating effects of age or sex on the association between psychosocial work conditions and health risks^13,17,18^. However, to the best of our knowledge, no study thus far has investigated the effects of psychological symptoms using the BSRS-5 and health status ratings measures on burnout in male and female HCWs of different age groups.

This study evaluates the potential associations of psychological symptoms assessed by BSRS-5 score, work characteristics assessed by a structured questionnaire, and health status ratings on the burnout levels assessed by CMBI-HSS of healthy mental subjects. We postulated that the BSRS-5 score, health status ratings, and work characteristics should be associated with burnout in terms of age and sex.

## Results

**Figure 1:**
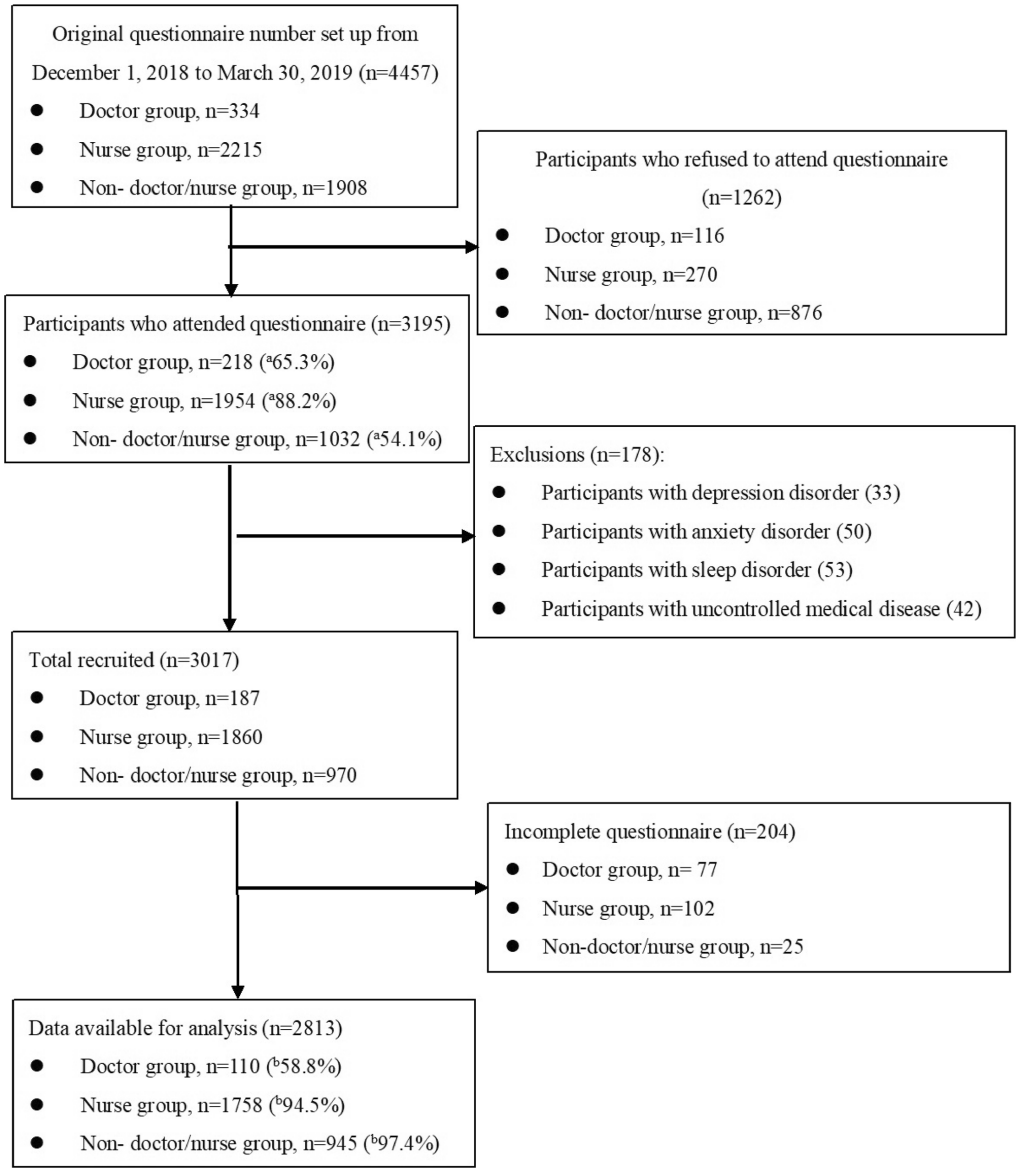
Selection Process of Participants. (**a**): Response rate. (**b**): Complete rate.

The potential participants were 4457 persons. 1262 staffs refused to joint it. Response rate of survey was 65.3% (doctor), 88.2% (nurse), and 54.1% (non-doctor/nurse). A total of 187 doctors, 1860 nurses, and 970 non-doctor/nurse participants were invited after performing the exclusion procedure (see Fig. 1), and 110, 1758, and 945 cases from the three groups completed the survey. The actual response rates were 58.8% (110/187) in the doctor group, 94.5% (1758/1860) in the nurse group, and 97.4% (945/970) in the non-doctor/nurse group. In total of 2813 participants were included in this study, implying a sufficient statistical power. Reliability of MBI-HSS and BSRS-5 questionnaire for all participants, stratified by sex and age was performed in Table 1. The results showed the internal consistency coefficients (Cronbach's α) of the MBI-HSS, and BSRS-5 were 0.79 (acceptable) and 0.89 (good), respectively (22–24) for all participants. Stratifying by sex and age, the Cronbach's α of MBI-HSS was more than 0.8 (good) for 60–79 years among both men and women, and between 0.7 and 0.8 (acceptable) for less than 60 years. The Cronbach's α of BSRS-5 score was more than 0.9 (excellent) for men among different age groups, and between 0.8 and 0.9 (good) for women among different age groups.

**Table 1:** Reliability of MBI-HSS and BSRS-5 questionnaire for all participants, stratified by sex and age.

Cronbach's alpha		
	MBI-HSS	BSRS-5
**Total**	0.79	0.89
**Men**		
20–39 Y/O	0.80	0.90
40–59 Y/O	0.76	0.91
60–79 Y/O	0.81	0.93
**Women**		
20–39 Y/O	0.79	0.89
40–59 Y/O	0.78	0.87
60–79 Y/O	0.84	0.89

BSRS-5 = 5-item Brief Symptom Rating Scale, MBI-HSS = Maslach Burnout Inventory–Health Services Survey.

This study included a total of 2582 non-burnout and 231 burnout participants. The prevalence of men in the burnout group was significantly higher than in the non-burnout group (15.22% vs. 10.19%, *p* = 0.02; Table 2). The college education level and exercise habits of the non-burnout group were significantly higher than those of the burnout group.

**Table 2:** Demographic and clinical characteristics of study participants across burnout situation.

Variables	Total (n = 2813)		No-burnout (n = 2582, 91.8%)		Burnout (n = 231, 8.2%)		*p* value
Age, years	38.44 ± 11.90		38.56 ± 11.99		37.08 ± 10.77		0.07
Men, n (%)	296	10.52%	261	10.11%	35	15.15%	0.02
BMI, kg/m^2^	23.43 ± 4.38		23.38 ± 4.34		23.99 ± 4.86		0.04
WC, cm	76.99 ± 10.83		76.89 ± 10.73		78.19 ± 11.80		0.08
Exercise, n (%)	1445	51.37%	1354	52.44%	91	39.39%	< 0.001
Smoking, n (%)	103	3.66%	94	3.64%	9	3.90%	0.84
Drink, n (%)	187	6.65%	168	6.51%	19	8.23%	0.32
HTN, n (%)	127	4.70%	109	4.39%	18	8.26%	0.01
DM, n (%)	75	2.77%	67	2.70%	8	3.67%	0.40
Hyperlipidemia, n (%)	71	2.63%	66	2.66%	5	2.29%	0.75
**Education level, n (%)**							0.04
≦Senior high school	371	13.19%	334	12.94%	37	16.02%	
College	2197	78.10%	2014	78.00%	183	79.22%	
≧ Graduate School	245	8.71%	234	9.06%	11	4.76%	
**Department**							0.13
Non-nurse/doctor	945	33.59%	859	33.27%	86	37.23%	
Nurse	1758	62.50%	1626	62.97%	132	57.14%	
Doctor	110	3.91%	97	3.76%	13	5.63%	
**Seniority, n (%)**							0.004
< 2 years	529	18.81%	486	18.82%	43	18.61%	
2–4 years	471	16.74%	428	16.58%	43	18.61%	
4–10 years	549	19.52%	487	18.86%	62	26.83%	
> 10yeras	1264	44.93%	1181	45.74%	83	35.93%	
**Working hours/week, n (%)**							< 0.001
≦ 45 h	1798	63.92%	1679	65.03%	119	51.52%	
46–50 h	795	28.26%	716	27.73%	79	34.20%	
51–59 h	129	4.59%	111	4.30%	18	7.79%	
≧ 60 h	91	3.23%	76	2.94%	15	6.49%	
**Work style, n (%)**							0.14
Day shift	1445	51.37%	1337	51.78%	108	46.75%	
Night shift	1368	48.63%	1245	48.22%	123	53.25%	
**BSRS-5, n (%)**							< 0.001
0–5	1805	64.17%	1751	67.82%	54	23.38%	
6–9	618	21.97%	549	21.26%	69	29.87%	
10–14	320	11.38%	241	9.33%	79	34.20%	
15–20	70	2.49%	41	1.59%	29	12.55%	
**Self-assessment physical health status, n (%)**							< 0.001
Good	827	29.40%	802	31.06%	25	10.82%	
Moderate	1720	61.14%	1574	60.96%	146	63.20%	
Poor	266	9.46%	206	7.98%	60	25.97%	
Sleep duration, hours (mean, SD)	6.58 ± 1.06		6.59 ± 1.05		6.44 ± 1.13		0.04
**Self-assessment sleep quality, n%**							< 0.001
Good	1118	39.74%	1062	41.13%	56	24.24%	
Poor	1695	60.26%	1520	58.87%	175	75.76%	
**MBI-HSS (mean, SD)**							
Emotional exhaustion	25.23 ± 10.86		24.06 ± 10.30		38.25 ± 8.16		< 0.001
Depersonalization	10.57 ± 5.86		9.92 ± 5.49		17.78 ± 4.86		< 0.001
Personal accomplishment	26.71 ± 5.45		27.30 ± 5.17		20.16 ± 3.90		< 0.001

n = numbers, SD = Standard deviation, HTN = hypertension, DM = diabetes mellitus, BMI = body mass index, WC = waist circumference, BSRS-5 = 5-item Brief Symptom Rating Scale, MBI-HSS = Maslach Burnout Inventory–Health Services Survey.

^†^Smoking status (current or past/never), alcohol consumption (0–1 drinks per week/≧ 2 drinks per week), exercise (≧ 3 times/week, ≧ 30 min/time).

Table 2 shows no statistical difference in terms of department and work style between the two groups. The seniority of the burnout group was less than ten years. A total of 45.8% of the non-burnout group had more than ten years of experience. The working hours of the burnout group exceeded those of the non-burnout group—approximately 48.5% of the burnout group reported working for more than 46 h per week. The BSRS-5 score ≥ 6 subgroup proportion was higher in the burnout group than in the non-burnout group (76.62% vs. 32.18%, *p* < 0.001). Sleep duration was shorter for the burnout group. Poor self-assessed physical health status and inadequate self-assessed sleep time were found in the burnout group (*p* < 0.001).

The participants’ data was divided into four categories of BSRS-5 scores, as shown in Table 3; younger age and women were found predominantly in the groups with higher BSRS-5 scores (*p* < 0.001 and *p* = 0.003). We also identified a significant trend of association between less exercise, drinking habit, and above college level education and higher BSRS-5 scores (Table 3).

**Table 3:** Demographic and clinical characteristics of study participants across different level of BSRS-5.

Variables	Total (n = 2813)	BSRS-5 score				ANOVA/chi-square test		Cochran–Armitage trend test	
		≦ 5	6–9	10–14	≧ 15				
		(n = 1805)	(n = 618)	(n = 320)	(n = 70)	Effect size test	*P* value	Effect size test	*P* value
Age, years	38.44 ± 11.90	39.81 ± 12.04	37.37 ± 11.57^a^	34.06 ± 10.50^a,b^	32.61 ± 9.58^a,b^	0.03	< 0.001	0.03	< 0.001
Men, n (%)	296 (10.5%)	219 (12.2%)	44 (7.2%)^a^	27 (8.5%)	6 (8.6%)	0.003	0.003	0.003	0.003
BMI, kg/m^2^	23.43 ± 4.38	23.44 ± 4.25	23.58 ± 4.50	23.06 ± 4.80	23.85 ± 4.75	0.001	0.30	< 0.001	0.66
WC, cm	76.99 ± 10.83	77.19 ± 10.55	77.02 ± 11.00	75.90 ± 11.67	76.74 ± 12.35	0.001	0.27	0.001	0.54
Exercise, n (%)	1445 (51.4%)	1011 (56.0%)	279 (45.1%)^a^	133 (41.6%)^a^	22 (31.4%)^a^	0.13	< 0.001	0.13	< 0.001
Smoking, n (%)	103 (3.7%)	67 (3.7%)	23 (3.7%)	12 (3.8%)	1 (1.4%)	0.02	0.80	0.02	0.63
Drink, n (%)	187 (6.6%)	107 (5.9%)	40 (6.5%)	30 (9.4%)	10 (14.3%)^a^	0.07	0.01	0.07	0.002
HTN, n (%)	127 (4.7%)	79 (4.6%)	30 (5.0%)	16 (5.2%)	2 (2.9%)	0.02	0.84	0.02	0.90
Diabetes, n (%)	75 (2.8%)	47 (2.7%)	16 (2.7%)	11 (3.5%)	1 (1.4%)	0.02	0.76	0.02	0.83
Hyperlipidemia, n (%)	71 (2.6%)	46 (2.7%)	18 (3.0%)	6 (1.9%)	1 (1.4%)	0.02	0.73	0.02	0.51
**Education level, n (%)**									
≦ Senior high school	371 (13.2%)	281 (15.6%)	62 (10.0%)^a^	23 (7.2%)^a^	5 (7.1%)				
College	2197 (78.1%)	1341 (74.3%)	505 (81.7%)^a^	289 (90.3%)^a, b^	62 (88.6%)^a^	0.14	< 0.001	0.14	0.34
≧ Graduate School	245 (8.7%)	183 (10.1%)	51 (8.3%)	8 (2.5%)^a^	3 (4.3%)				
**Department, n (%)**									
Non-nurse/doctor	945 (33.6%)	706 (39.1%)	162 (26.2%)^a^	68 (21.3%)^a^	9 (12.9%)^a^				
Nurse	1758 (62.5%)	1021 (56.6%)	435 (70.4%)^a^	242 (75.6%)^a^	60 (85.7%)^a, b^	0.17	< 0.001	0.17	< 0.001
Doctor	110 (3.9%)	78 (4.3%)	21 (3.4%)	10 (3.1%)	1 (1.4%)				
**Seniority, n (%)**									
< 2 years	529 (18.8%)	330 (18.3%)	101 (16.3%)	81 (25.3%)^a^	17 (24.3%)				
2–4 years	471 (16.7%)	298 (16.5%)	109 (17.6%)	51 (15.9%)	13 (18.6%)				
4–10 years	549 (19.5%)	332 (18.4%)	127 (20.6%)	69 (21.6%)	21 (30.0%)	0.10	0.001	0.10	0.001
> 10yeras	1264 (44.9%)	845 (46.8%)	281 (45.5%)	119 (37.2%)^a^	19 (27.1%)^a, b^				
**Working hours/week, n (%)**									
≦ 45 h	1798 (63.9%)	1239 (68.7%)	360 (58.3%)^a^	171 (53.4%)^a^	28 (40.0%)^a, b^				
46–50 h	795 (28.2%)	454 (25.1%)	200 (32.4%)^a^	111 (34.7%)^a^	30 (42.9%)^a^	0.15	< 0.001	0.15	< 0.001
51–59 h	129 (4.6%)	61 (3.4%)	35 (5.7%)	25 (7.8%)^a^	8 (11.4%)^a^				
≧ 60 h	91 (3.3%)	51 (2.8%)	23 (3.7%)	13 (4.1%)	4 (5.7%)				
**Work style, n (%)**									
Regular class	1445 (51.4%)	1029 (57.0%)	282 (45.6%)^a^	114 (35.6%)^a, b^	20 (28.6%)^a, b^				
Night shift	1368 (48.6%)	776 (43.0%)	336 (54.4%)^a^	206 (64.4%)^a, b^	50 (71.4%)^a, b^	0.17	< 0.001	0.17	< 0.001
**Self-assessment physical health status, n (%)**									
Good	827 (29.4%)	694 (38.4%)	92 (14.9%)^a^	36 (11.3%)^a^	5 (7.1%)^a^				
Common	1720 (61.1%)	1033 (57.2%)	447 (72.3%) ^a^	216 (67.5%) ^a^	24 (34.3%) ^a, b, c^	0.40	< 0.001	0.40	< 0.001
Bad	266 (9.5%)	78 (4.3%)	79 (12.80%) ^a^	68 (21.3%) ^a, b^	41 (58.6%) ^a,b,c^				
Sleep duration, hours (mean, SD)	6.58 ± 1.06	6.69 ± 1.01	6.44 ± 1.11^a^	6.37 ± 1.09 ^a^	5.98 ± 1.15 ^a, b, c^	0.02	< 0.001	0.02	< 0.001
**Self-assessment of sleep quality, n (%)**									
Good	1118 (39.7%)	873 (48.4%)	172 (27.8%)^a^	67 (20.9%)^a^	6 (8.6%)^a, b^				
Poor	1695 (60.3%)	932 (51.6%)	446 (72.2%)^a^	253 (79.1%)^a^	64 (91.4%)^a, b^	0.24	< 0.001	0.24	< 0.001
Burnout, n (%)	231 (8.2%)	54 (3.0%)	69 (11.2%)^a^	79 (24.7%)^a, b^	29 (41.4%)^a, b, c^	0.32	< 0.001	0.32	< 0.001

^a^*p* < 0.05 versus BSRS-5 score ≦ 5 group.

^b^*p* < 0.05 versus BSRS-5 score 6–9 group.

^c^*p* < 0.05 versus BSRS-5 score 10–14 group in the Bonferroni post hoc comparisons.

BSRS-5: 5-item Brief Symptom Rating Scale, n = numbers, SD = Standard deviation, HTN = hypertension, DM = diabetes mellitus, BMI = body mass index, WC = waist circumference.

**Figure 2:**
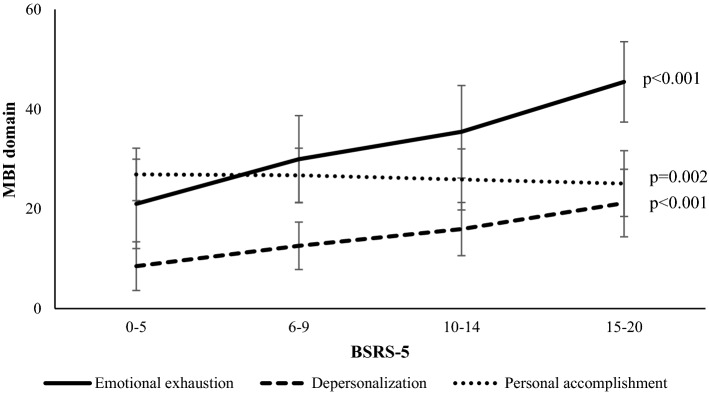
BSRS-5 severity and mean score on the domains of Maslach Burnout Inventory (MBI). Note. The graph shows mean burnout domains (assessed with the Maslach Burnout Inventory) as a function of the level of psychological symptoms (± 1 standard deviation [SD]), assessed with the 5-item Brief Symptom Rating Scale (BSRS-5). BSRS-5 scores of 0–5, 6–9, 10–14, and 15–20 reflect the cutoffs for normal, mild, moderate, and severe psychological symptoms, respectively^39,47^. The vertical lines represent the standard deviations (.77 < SD < .99) of the burnout domain score at each level of psychological symptoms severity. Note: BSRS-5: 5-item Brief Symptom Rating Scale; MBI: Maslach Burnout Inventory.

The seniority of the higher BSRS-5 scores was primarily 4–10 years. We also found a significant decreasing trend of association between seniority of more than 10 years and higher BSRS-5 scores. Similarly, working more than 46 h per week and night shifts were related to higher BSRS-5 scores. There was a significant trend of association between shorter sleep duration, poor self-assessed physical health status, poor self-assessed sleep quality, and higher burnout prevalence and higher BSRS-5 scores (*p* < 0.001). Medium to high effect size test in ANOVA/chi square test was in self-assessment physical health status and burnout. Medium to high effect size test in Cochran–Armitage Trend test was exercise, drinking, education, and seniority; high effect size test in Cochran–Armitage Trend test was department, working hours/week, work style, s self-assessment physical health status, self-assessment of sleep time, and burnout. Figure 2 shows the relationship between the three domains of the MBI-HSS scores and the four categories of the BSRS-5 scores (*p* < 0.05).

Table 4A shows that EE and DP have a good correlation with anxiety, depression, and total BSRS-5 scores (r = 0.5–0.75, *p* < 0.001); however, PA was irrelevant by Spearman’s correlation. Table 4B showed that EE and DP had good correlation with all items except poor sleep quality among men less than 30 years, DP had good correlation with anxiety, inferiority, and total BSRS-5 score among men more than 30 years. EE and DP had a good correlation with anxiety, depression, hostility, and total BSRS-5 scores among women with different age group. Supplement Table 2 showed the similar results by Pearson correlation. The results showed a higher prevalence of burnout among 20–39 years old women with a BSRS-5 score > 6 or the health status ratings measure ≥ 1 (Table 5).

**Table 4:** A. Correlation between the items of BSRS-5 and domain of MBI, B. Correlation between the items of BSRS-5 and domain of MBI stratified by age and gender.

Variables	Emotional exhaustion		Depersonalization		Personal accomplishment	
	Spearman's correlation coefficient	*p* value	Spearman's correlation coefficient	*p* value	Spearman's correlation coefficient	*p* value
BSRS-5 (5 item)						
Anxiety^1^	0.54	< 0.001	0.55	< 0.001	− 0.01	0.50
Depression^2^	0.60	< 0.001	0.55	< 0.001	− 0.07	< 0.001
Hostility^3^	0.59	< 0.001	0.54	< 0.001	− 0.05	0.01
Inferiority^4^	0.46	< 0.001	0.50	< 0.001	− 0.07	< 0.001
Trouble falling asleep^5^	0.46	< 0.001	0.38	< 0.001	− 0.05	0.01
BSRS-5 score	0.65	< 0.001	0.61	< 0.001	− 0.06	0.002
**Maslach Burnout Inventory (MBI)**						
**Men < 30 years**						
BSRS-5 (5 item)						
Anxiety	0.68	< 0.001	0.59	< 0.001	− 0.18	0.13
Depression	0.68	< 0.001	0.61	< 0.001	− 0.20	0.10
Hostility	0.72	< 0.001	0.63	< 0.001	− 0.36	0.002
Inferiority	0.58	< 0.001	0.61	< 0.001	− 0.13	0.28
Trouble falling asleep	0.57	< 0.001	0.48	< 0.001	− 0.15	0.21
BSRS-5 score	0.75	< 0.001	0.67	< 0.001	− 0.22	0.07
**Men ≧ 30 years**						
BSRS-5 (5 item)						
Anxiety	0.55	< 0.001	0.56	< 0.001	− 0.13	0.05
Depression	0.62	< 0.001	0.47	< 0.001	− 0.15	0.03
Hostility	0.57	< 0.001	0.44	< 0.001	− 0.10	0.15
Inferiority	0.52	< 0.001	0.52	< 0.001	− 0.25	< 0.001
Trouble falling asleep	0.45	< 0.001	0.39	< 0.001	− 0.18	0.01
BSRS-5 score	0.65	< 0.001	0.56	< 0.001	− 0.19	0.01
**Women < 30 years**						
BSRS-5 (5 item)						
Anxiety	0.49	< 0.001	0.51	< 0.001	0.004	0.90
Depression	0.59	< 0.001	0.57	< 0.001	− 0.06	0.10
Hostility	0.60	< 0.001	0.53	< 0.001	− 0.07	0.03
Inferiority	0.44	< 0.001	0.50	< 0.001	− 0.05	0.11
Trouble falling asleep	0.46	< 0.001	0.40	< 0.001	− 0.03	0.34
BSRS-5 score	0.62	< 0.001	0.60	< 0.001	− 0.05	0.15
**Women ≧ 30 years**						
BSRS-5 (5 item)						
Anxiety	0.55	< 0.001	0.55	< 0.001	− 0.02	0.41
Depression	0.59	< 0.001	0.53	< 0.001	− 0.09	0.001
Hostility	0.58	< 0.001	0.55	< 0.001	− 0.03	0.29
Inferiority	0.45	< 0.001	0.49	< 0.001	− 0.07	0.01
Trouble falling asleep	0.44	< 0.001	0.36	< 0.001	− 0.05	0.04
BSRS-5 score	0.64	< 0.001	0.60	< 0.001	− 0.06	0.02

BSRS-5 = 5-item Brief Symptom Rating Scale, MBI = Maslach Burnout Inventory.

Ref:^39–41,48^.

**Table 5:** The association between level of BSRS-5 score and health status ratings with the prevalence of burnout by using chi square test stratified by age and gender.

Age	20–39 Y/O		40–59 Y/O		60–79 Y/O		*p* value	p trend
**BSRS-5 score**								
Men	(n = 165, 55.74%)		(n = 116, 39.19%)		(n = 15, 5.07%)		0.77	0.22
0–5	118	71.76%	92	79.20%	11	75.00%		
6–9	27	16.38%	16	13.60%	2	12.50%		
10–14	16	9.60%	6	5.60%	2	12.50%		
15–20	4	2.26%	2	1.60%	0	0.00%		
Women	(n = 1387, 55.11%)		(n = 1040, 41.32%)		(n = 90, 3.57%)		< 0.001	< 0.001
0–5	792	57.09%	709	68.19%	80	88.76%		
6–9	336	24.22%	231	22.21%	8	9.00%		
10–14	210	15.13%	86	8.24%	1	1.12%		
15–20	49	3.56%	14	1.36%	1	1.12%		
**Health status ratings**								
Men							0.87	0.76
0	75	45.76%	54	46.40%	8	56.25%		
1	78	46.89%	51	44.00%	6	37.50%		
2	12	7.35%	11	9.60%	1	6.25%		
Women							< 0.001	< 0.001
0	461	33.24%	427	41.06%	45	49.44%		
1	796	57.38%	549	52.82%	43	48.31%		
2	130	9.38%	64	6.12%	2	2.25%		

BSRS-5 = 5-item Brief Symptom Rating Scale.

Definition: Health status ratings included 1. self-assessment physical health status (good, common, and poor); and 2. self-assessment sleep quality (good and poor).

0 means the answers included self-assessment physical health status (good or moderate) and self-assessment sleep quality (good).

1 means the answers included self-assessment physical health status (good or moderate) and self-assessment sleep quality (poor); self-assessment physical health status (poor) or self-assessment sleep quality (good).

2 means the answers included self-assessment physical health status (poor) and self-assessment sleep quality (poor).

The logistic regression analysis was performed to estimate the crude and multivariable-adjusted odds (OR) ratios and identify factors associated with burnout (Table 6). The BSRS-5 scores, age, sex, and factors (working characters and health status ratings that were significantly associated with burnout in crude analysis were included in the multiple logistic regression model. The BSRS-5 scores (compared to 0–5, OR of 6–9, 10–14, and 15–20 = 1.83, 3.23, 9.15, respectively; p < 0.01) and being a man (OR = 1.88, 95% confidence interval [CI] = 1.15–3.09) were still associated with burnout, whereas the work characteristics that remained significant included a seniority of 2–4 years and 4–10 years (OR = 1.79, 1.69, p < 0.05), poor self-assessed health status (OR = 3.31, 95% CI 1.84–5.97), and poor self-assessed sleep quality (OR = 1.78, 95% CI 1.08–2.23), and were significantly associated with burnout.

**Table 6:** Univariate and multivariate logistic regression analysis of factors influencing burnout.

Variables	Crude			Multi-variable		
	OR	95% CI	*p* value	OR	95% CI	*p* value
**BSRS-5**						
0–5	1	–	–	1	–	–
6–9	1.98	(1.36–2.90)	< 0.001	1.83	(1.23–2.72)	0.003
10–14	3.44	(2.29–5.16)	< 0.001	3.23	(2.08–5.01)	< 0.001
15–20	12.56	(7.27–21.70)	< 0.001	9.15	(4.90–17.10)	< 0.001
Age	0.99	(0.98–1.00)	0.07	1.01	(0.99–1.02)	0.29
Men	1.58	(1.08–2.32)	0.02	1.88	(1.15–3.09)	0.01
BMI	1.03	(1.00–1.06)	0.04	1.02	(0.99–1.06)	0.19
WC	1.01	(0.99–1.02)	0.08	–	–	–
Exercise (no versus yes)	1.70	(1.29–2.23)	< 0.001	0.77	(0.57–1.06)	0.11
Smoking (yes vs. no)	1.07	(0.53–2.16)	0.84	–	–	–
Drink (yes vs. no)	1.29	(0.79–2.11)	0.32	–	–	–
HTN (yes vs. no)	1.96	(1.17–3.30)	0.01	1.64	(0.88–3.07)	0.12
DM (yes vs. no)	1.37	(0.65–2.90)	0.40	–	–	–
Hyperlipidemia (yes vs. no)	0.86	(0.34–2.16)	0.75			
**Education level**						
≦Senior high school	1	–	–	–	–	–
College	0.82	(0.57–1.19)	0.30	–	–	–
≧Graduate School	0.42	(0.21–0.85)	0.02	–	–	–
**Department**						
Non-doctor/nurse	1	–	–	–	–	–
Nurse	1.81	(0.93–2.77)	0.25	–	–	–
Doctor	1.34	(0.72–2.49)	0.36	–	–	–
**Seniority**						
< 2 years	1	–	–	1	–	–
2–4 years	1.79	(1.06–3.05)	0.03	1.79	(1.03–3.11)	0.04
4–10 years	1.97	(1.19–3.27)	0.01	1.69	(1.01–2.88)	0.03
> 10 yeras	1.33	(0.83–2.13)	0.24	0.88	(0.48–1.59)	0.67
**Working hours/week**						
≦ 45 h	1	–	–	1	–	–
46–50 h	1.56	(1.16–2.10)	0.003	1.19	(0.86–1.66)	0.29
51–59 h	2.29	(1.34–3.89)	0.002	1.17	(0.62–2.21)	0.62
≧ 60 h	2.78	(1.55–4.99)	0.001	1.28	(0.49–3.34)	0.61
**Work style**						
Day shift	1	–	–	–	–	–
Night shift	1.22	(0.93–1.60)	0.14	–	–	–
**Self-assessment physical health status**						
Good	1	–	–	1	–	–
Moderate	2.98	(1.93–4.59)	< 0.001	2.11	(1.28–3.48)	0.003
Poor	9.34	(5.72–15.27)	< 0.001	3.31	(1.84–5.97)	< 0.001
Sleep duration, hours	0.87	(0.77–0.99)	0.04	1.11	(0.97–1.28)	0.14
**Self-assessment sleep quality**						
Good	1	–	–	–	–	–
Poor	2.18	(1.60–2.98)	< 0.001	1.78	(1.08–2.23)	0.004

OR = Odds Ratio; CI Confidence interval.

Multivariable model included: BSRS-5, age, gender and independence of risk factors in the univariate analysis.

After stratifying age and sex (Table 7), the results suggested that men ≥ 30 years of age and women of all age groups with a BSRS-5 score ≥ 6 (men, OR = 4.55, 95% CI 1.66–12.45; women, OR = 2.05 and 3.40 for different age groups; *p* < 0.05); women with longer working hours (more than 46 h/week, *p* < 0.05); men and women (both ≥ 30 years of age) with night-shift jobs (men, OR = 3.27, 95% CI 1.12–9.61; women, OR = 1.72, 95% CI 1.18–2.53); poor self- assessed health status (men, OR = 4.00 and 7.37; women, OR = 4.75 and 4.88 for different age groups; *p* < 0.05); and poor self-assessed sleep quality (men, OR = 4.20 and 3.30; women, OR = 2.20 and 1.77 for different age group; *p* < 0.05) were highly associated with burnout. However, there was no statistical difference in the relevance of burnout among various departments, including the doctor, nurse, and non-doctor/nurse groups.

**Table 7:** The association between BSRS-5, working character, and perception of wellness and burnout by using multivariate logistic regression analysis stratified by age and sex.

	Men (296, 10.5%)						Women (2517, 89.5%)					
	< 30 years (n = 69, 23.3%)			≧ 30 years (n = 227, 76.7%)			< 30 years (n = 863, 34.3%)			≧3 0 years (n = 1654, 65.7%)		
	OR	(95% CI)	*p* value	OR	(95% CI)	*p* value	OR	(95% CI)	*p* value	OR	(95% CI)	*p* value
**BSRS-5 score**												
< 6	1			1			1			1		
≧ 6	1.55	(0.54–2.32)	0.06	4.55	(1.66–12.45)	0.003	2.05	(1.12–3.74)	0.02	3.40	(2.30–5.03)	< 0.001
**Department**												
Non-nurse/doctor	1			1			1			1		
Nurse	0.62	(0.10–4.01)	0.61	0.75	(0.16–3.59)	0.71	1.11	(0.43–2.88)	0.83	1.10	(0.74–1.63)	0.63
Doctor	1.11	(0.34–3.56)	0.84	2.14	(0.72–6.34)	0.17	1.73	(0.18–16.16)	0.63	1.98	(0.23–4.23)	0.97
**Working hours/week**												
≦ 45 h	1			1			1			1		
46–50 h	4.62	(0.48–43.94)	0.18	0.54	(0.11–2.57)	0.44	2.59	(1.32–5.08)	0.01	1.19	(0.77–1.84)	0.44
51–59 h	0.00			2.46	(0.48–12.72)	0.28	5.22	(2.11–12.95)	< 0.001	2.90	(1.37–6.16)	0.01
≧ 60 h	0.00			2.27	(0.65–7.94)	0.20	2.61	(1.05–8.07)	< 0.001	1.19	(1.02–5.11)	0.04
**Work style**												
Day shift	1			1			1			1		
Night shift	1.87	(0.20–17.75)	0.58	3.27	(1.12–9.61)	0.03	2.32	(0.91–5.96)	0.08	1.72	(1.18–2.53)	0.01
**Self-assessment physical health status**												
Good	1			1			1			1		
Moderate	2.40	(0.21–27.78)	0.48	2.03	(0.53–7.73)	0.30	2.29	(1.32–3.96)	0.003	1.10	(0.49–2.43)	0.82
Poor	4.00	(1.66–7.89)	0.02	7.37	(1.62–33.54)	0.01	4.75	(2.37–9.53)	< 0.001	4.88	(2.10–11.35)	< 0.001
**Self-assessment sleep quality**												
Good	1			1			1			1		
Poor	4.20	(1.18–8.14)	0.04	3.30	(1.13–5.68)	0.01	2.20	(1.08–4.48)	0.03	1.77	(1.16–2.70)	0.01

Adjusted factors: body mass index, waist circumference, personal habits, chronic diseases, and education level.

## Discussion

To the best of our knowledge, this is the first study to demonstrate that the severity of psychological symptoms, measured by the BSRS-5, can significantly associated with burnout among different sex and age, and that this effect persists after controlling for common potential confounders, including age, sex, smoking, alcohol consumption, exercise, and chronic diseases (HTN, DM, and hyperlipidemia). We also performed the stratified analysis to identify the association of high workload and poor health status ratings with burnout among middle-aged men and women.

The result showed that the prevalence of burnout among the entire sample population, men, and women was 8.2%, 11.8% and 7.8%, respectively (*p* = 0.04). Compared with other studies^1,2,4,5^, the possible reasons for the lower incidence of burnout found in this study included different departments including in this study, not only physicians and nurses. Second, the participants in this study were generally younger than those in other studies (median 38.4 years)^19–21^. Finally, 86.8% of the participants in this study possessed a higher level of education (higher than university) as compared to participants in other studies^19–21^. A previous study described a high risk of burnout mostly among women with low education levels^22^.

Burnout, SRH and psychologic symptoms are not consistent with age and gender in HCWs in different departments^12,13,23,24^. Previous study had clarified that age and sex are a potential modulator of health risks caused by psychosocial work conditions^25^. Surveys of physicians from different specialties found that burnout was self-reported had found that women were more likely to exhibit burnout symptoms than were men^26–28^. Age was an independent variable associated with burnout, with younger physicians at a higher risk of burnout than older physicians^29^. In previous studies mentioned that it was also worth noting that age may influence burnout more than gender did^27^.

Most of previous studies are based on different indicators to discuss age or sex stratification^13,21,24,29^. Few studies have combined psychological symptoms, health status ratings, age, and sex into groups to explore the correlation with burnout. This study shows that whether men and women are over 30 with a high BSRS-5 score or poor health status ratings is higher than that of people under 30 years old. This echoes with previous studies of individual indicators, these previous results show that poor SRH increases with age, and the level of burnout among employees aged 30–40 is higher than that of other age groups^11,13^.

In terms of sex, previous studies proposed that burnout symptoms vary considerably according to the different life stages of working men and women^28,30^. Their research found that two groups including aged 20–35 years and aged 55 years and above were particularly susceptible to burnout, and that different psychosocial conditions needed to be considered. Therefore, in our study, we included different psychosocial factors (such as BSRS-5 and health status ratings) that caused different levels of burnout among men and women of different age groups.

Studies have mentioned that physical and mental diseases affect psychological symptoms and burnout^15,31^. We excluded participants with uncontrolled chronic diseases and mental and sleep disorders. However, we found that some under-treatment chronic diseases (HTN, DM, hyperlipidemia) did not cause burnout [HTN: 1.64 (0.88–3.07); DM: 1.37 (0.65–2.90), and hyperlipidemia: 0.86 (0.34–2.16)].

Recent research has stated that poor sleep quality is common, and associated with increased burnout, among clinicians delivering care to COVID-19 patients^32^. In this model, self-reported poor sleep quality was significantly associated with burnout (OR = 4.13, 95% CI 2.33–7.32, *p* < 0.05), whereas short sleep duration (< 6 h) was not (OR = 0.73, 95% CI 0.41–1.30, *p* = 0.28). Interestingly, we found that sleep quality was more strongly related to burnout than sleep duration, even during the non-pandemic period (longer sleep time [OR = 1.11, 95% CI 0.97–1.28, *p* = 0.14] and poor self-assessed sleep quality [OR = 1.78, 95% CI 1.08–2.23, *p* = 0.004]).

As age increases, physical and mental health susceptibility increases. Stressful work conditions, such as long hours of work and heavy workload, may induce greater physiological responses in older workers^13^. Our study shows that middle-aged/elderly HCWs with longer working hours and night shifts exhibit greater physical and psychological reactions than younger staff, especially among women. Among the work characteristics, longer working hours (more than 46 h/week) for women and night shift jobs for both men and women aged 30 years or above were found to be the most common predictors of high burnout. These findings support the results of a previous study conducted in Taiwan and other countries^13,33–35^.

Pappas et al. found that nurses in Greece with poor SRH were significantly more likely to be female, between the ages of 30–39 years, have long working hours, and rotating shifts^36^. The impact of poor self-assessed sleep quality on men and women younger than 30 years was more significant than on those older than 30 years. The OR for men was higher than that for women (men, OR: < 30 years of age vs.  ≥ 30 years of age = 4.20:3.30; women, OR: < 30 years of age vs.  ≥ 30 years of age = 2.20:1.77; all, *p* < 0.05). Our research has the same findings for HCWs as the study by Pappas et al. The results of our research and previous studies on hospital workplaces^37^ are inconsistent with previous results in the general population^37,38^. This may be due to the influence of the work characteristics.

Although burnout, anxiety, and depression have many overlapping issues, Fischer et al. focused on 715 critical care clinicians in Brazil and, using appropriate statistical methods, they found that burnout was statistically distinct from anxiety and depression^15^. These findings suggest that health professionals at high risk of stress need to be screened for both burnout and clinical psychological symptoms to provide timely and efficient treatment. Even the BSRS-5 is not a generic questionnaire and is not commonly used in burnout studies. The reasons why we chose it for the measurement tool first: it contains only five items and can be administered to subjects to complete independently and quickly. This questionnaire is already recommended to the general public to detect psychological symptoms and suicide prevention in Taiwan^39–41^. Second: good reliability of MBI-HSS and BSRS-5 was checked among all participants in our study (Table 1). This study provides additional evidence that may be suitable for the evaluation of psychological symptoms in HCWs with burnout syndrome.

The strengths of our study are as follows: first, we use simple BSRS-5 score and health status ratings problems to reveal the association and evaluate the burnout problem among HCWs. We also performed age and sex stratification to facilitate burnout prevention in high-risk HCWs of a particular age and sex. Second, we excluded participants with untreated chronic diseases and mental and sleep disorders to prevent any interference with the results for burnout.

Alternatively, this study had some limitations. First, nearly 90% of the participants were women (men = 296, 10.5%; women = 2517, 89.5%). Although the chi-square test showed significant differences between the proportions of men and women for the burnout categories and levels of the BSRS-5 scores, there were some restrictions in the case of men when stratifying analysis by age and sex because of the small sample size (men: < 30 years of age, n = 69; ≥ 30 years of age, n = 227). Due to the absence of measurement of social desirability, in the future, data should be collected from multiple hospitals and regions, and studies should include more male participants. Second, this study was cross-sectional, and it was insufficient to confirm the causes and effects of psychological symptoms and burnout. Third, this study did not discuss family-related factors, including the pressure and time spending of taking care of children, organizing housework, etc., which have made the gender-related differences between work and non-work stressors. Fourth, we evaluated health status ratings by asking two questions including two items (physical health condition and sleep quality) because of the limitation related to the total number of items in the questionnaire; we did not use the more common “Perceived Wellness Survey,” which comprises 36 items^42^. Finally, this study was a voluntary response sample, and there was a problem of self-selection bias.

The BSRS-5 score is associated with the scores of at least two domains of the Chinese version of MBI-HSS for HCWs, whereas mild physical illness (such as HTN, DM, and hyperlipidemia) may not influence the results in the case of some participants. Researchers and health practitioners should pay attention to the influence of age and sex when using the measures of the BSRS-5, health status ratings, and burnout as indicators to detect health risks associated with adverse psychosocial work conditions.

## Methods

### Ethics

The study protocol was evaluated and approved by the Human Research Ethics Committee of the Mackay Memorial Hospital (project research number 18MMHISO150, 22. Nov.2018-21. Nov.2020). All participants provided written informed consent. Data confidentiality was preserved, considering ethical issues, such as autonomy and respect for people’s privacy, and all the guidelines of the Declaration of Helsinki were followed. To ensure data confidentiality, participant identification information was replaced with a folio number.

### Study population

This cross-sectional study was conducted at the Occupation Safety and Health Department of the Mackay Memorial Hospital, a 2000-bed tertiary teaching center in both the Taipei and New Taipei, Taiwan branch. This region has an estimated population of 2.67 million^43^. Workforces in all departments, such as doctors, nurses, technicians, administrative staff, pharmacists, radiologists, and nutritionists, were included, they were categorized as into the doctor, the nurse group, and the remaining staff were categorized as the non-doctors/nurse groups for the analysis*.* This study was a voluntary response sample. Questionnaires were administered between December 2018 and March 2019. The total number of questionnaires issued is 334 for doctors, 2215 for nurses, and 1908 for non-doctor/nurse participants. A sample size of 2533 achieved 90% power using the two tailed test (OR = 1.59, probability of null hypothesis = 0.15, alpha error = 0.05, power = 0.9, and R^2^ for other confounding factors = 0.5)^44^.

### Socio-demographic data and past medical history

Information was collected using structured questionnaires, designed according to the instructions of the Institute of Labor, Occupational Safety, Ministry of Labor (Survey of Perceptions of Safety and Health in the Work Environment in 2016 Taiwan; ILOSH105-M309)^45^. The following data were collected: socioeconomic status, work characteristics, job burnout syndrome status, and mental health status. Analysis of the internal consistency reliability of each part of the scale showed Cronbach's α to be between 0.82 and 0.93, indicating good reliability. The age group was divided to young adult (20–39 years old), middle-aged adult (40–59 years old), and old adult (60–79 years old)^46^. According to distribution, we divided into < 30 Y/O and ≧ 30 Y/O”.

The presence of hypertension (HTN), diabetes mellitus (DM), and hyperlipidemia was defined as a previous diagnosis of diseases or current medication use for these diseases.

### Baseline anthropometric measurements

Baseline characteristics and anthropometric measurements, including age, body height, body weight, body mass index, and waist circumference (WC), were recorded. Height was measured to the nearest 0.01 cm using a standard stadiometer. Weight was measured in light clothes to the nearest 0.01 kg using a set of standard calibrated electronic scales. The WC was measured using a constant-tension tape at the midpoint between the lowest rib and the upper point of the iliac crest, and at the end of normal expiration. Anthropometric measurements such as height, weight, and WC were examined and recorded by trained nurses who were blinded to the patients’ information.

### Work characteristics

Basic work-related issues included department and seniority. Information about working hours and shift jobs was derived from the work status provided by participants during the month before the survey. A night shift was described as that which encompassed midnight; work shifts that began in the morning, afternoon, or evening but did not extend to midnight were classified as day shifts. The participants were categorized into the following: day shift (day fixed/rotation) and night shift (night fixed/rotation)^47^.

### Burnout assessment

Burnout domains were evaluated using the Chinese version of the Maslach Burnout Inventory–Health Services Survey test (MBI-HSS) as per Lu et al. (Cronbach's α, 0.84)^48^. Three domains of the MBI-HSS were evaluated: emotional exhaustion (EE), depersonalization (DP), and personal accomplishment (PA) (Supplement Table 1). The MBI-HSS includes 22 questions in total, and the frequency of occurrence is scored using a 7-point Likert scale from 0 (never felt) to 6 (felt every day). According to the classification standard of previous study^48^, if EE score ≧ 27, DP score ≧ 13, and PA ≦ 31 was categorized as high job burnout; if EE score is between 17 and 26, DP score is between 7 and 12, and PA score is between 32 and 38, it is considered to be moderate job burnout; if EE score ≦ 16, DP score ≦ 6, and PA score ≧ 39 are considered low job burnout. The psychological work demands, EE and DP showed a direct relation to burnout, whereas PA showed a negative association^4^. The internal consistency coefficients (Cronbach's α) of the EE, DP, and PA dimensions were 0.77, 0.83, and 0.82, respectively^48^.

### Assessment of psychological symptoms: the 5-item Brief Symptom Rating Scale

The BSRS-5 contains five items of psychological symptoms and is commonly used for screening psychological disorders. It is available in Taiwan with excellent validity and reliability^40,41^ (Supplement Table 1). It is a self-administered questionnaire that measures anxiety (feeling tense or high-strung), depression (feeling depressed or in a low mood), hostility (feeling easily annoyed or irritated), interpersonal sensitivity (feeling inferior to others), and additional symptoms (having trouble falling asleep) during the past week. Responses are rated on a scale of 0–4 (0 = not at all; 1 = a little bit; 2 = moderately; 3 = quite a bit; and 4 = extremely). The total score ranges from 0 to 20^39^. When a score of ≥ 6 was used as the cutoff point for psychiatric cases, the BSRS-5 was divided into four groups: “no symptoms” (0–5), “mild” (6–9), “moderate” (10–14), and “severe” (> 15)^40^.

The internal consistency was analyzed using Cronbach's α coefficient: the BSRS-5 coefficients ranged from 0.77 to 0.90 (20), and the test–retest reliability coefficient was 0.82. Using a score of ≥ 6 as the cutoff point for psychiatric cases, the BSRS-5 accurate classification rate was 76.3% (sensitivity 78.9%; specificity 74.3%; positive predictive value 69.9%; and negative predictive value, 82.3%)^40^.

### Measure of health status: health status ratings

Health status ratings was assessed using two-item questions for physical health and sleep quality issues. The physical issue question was: “In general, how is your health?” it had three possible answers ranging: “good,” “moderate,” or “poor.” In case of the sleep quality issue, which was mentioned in the literature^6,13^: “How about your sleep quality?” which had two possible answers: “good” and “poor”^13^.

The health status ratings levels were divided into answers of 0, 1, and 2; their definitions are as follows: 0 means that the answers included self-assessed physical health status as good or moderate and self-assessed sleep quality as good; 1 means the answers included self-assessed physical health status as common or moderate or self-assessed sleep quality as poor; self-assessed physical health status as poor or self-assessed sleep quality as good; and 2 means the answers included self-assessed health status as poor and self-assessed sleep quality as poor.

### Statistical analysis

In this study, the findings of screening for the BSRS-5 scores and health status ratings level for healthcare professionals at high risk of burnout was primary outcome; and stratification analysis for age and sex was second outcome. Data are summarized as means and standard deviations for continuous variables or frequencies and percentages for categorical variables. The distribution of burnout and non-burnout syndrome according to basic characteristics was analyzed using chi-square tests for categorical variables and Student’s t-tests for continuous variables. We conducted an analysis of variance and chi-square tests for evaluating differences among variables; the Cochran–Armitage test for trend and the Bonferroni post hoc comparisons were performed for the participants, who were divided into four groups according to BSRS-5 score intervals of 0–5, 6–9, 10–14, and 15–20. Effect size, including Cohen’s W for chi-square tests and eta squared for ANOVA, was performed as a quantitative measure of the magnitude of the experimental effect^43^. The larger the effect size the stronger the relationship between two variables. Based on the rules of thumb^49^, we categorized the magnitudes of effect sizes into small, medium, and large.

Spearman’s correlation and Pearson correlation were performed between the five items of the BSRS-5 and the three domains of burnout^39,48,50^. Pearson correlation measures the "linear" correlation. The three domains of MBI-HSS and BSRS-5 in this study are “non-linear” variables, which is the main reason for adopting Spearman’s correlation in this study. Stratifying by sex and age was performed. The univariate logistic regression analyses were performed to investigate the possible associations between burnout and the BSRS-5, work-related factors, psycho-physical symptoms, and chronic disease status. Thereafter, the multivariate logistic regression analyses were performed in order to adjust for age, sex, and other variables that showed significant associations in the univariate analyses. Finally, we evaluated the association between the BSRS-5, work characteristics, perception of wellness, and burnout in the stratum of age and sex using the logistic regression analysis. All analyses were performed using SPSS 22 (IBM Corp., Armonk, NY, USA) for Windows. All tests were 2- tailed and *p* values of < 0.05 were considered statistically significant.

## Supplementary Information

Supplementary Information.
