# Supplementary material for: Association of 5-item Brief Symptom Rating Scale scores and health status ratings with burnout among healthcare workers
Source: Sci Rep. 2022 May 3;12:7122. doi: 10.1038/s41598-022-11326-1 (PMC9062289; doi:10.1038/s41598-022-11326-1)
Supplement: Supplementary file 1 — Supplementary Information. [file 41598_2022_11326_MOESM1_ESM.docx]

Supplement table 1 MBI-HSS and BSRS-5 items

| MBI-HSS (22 items) |  |
| --- | --- |
| Emotional exhaustion (EE) (9 items) | 1. I feel emotionally drained from my work. |
|  | 1. I feel used up at the end of the workday. |
|  | 1. I feel fatigued when I get up in the morning and have to face another day on the job. |
|  | 1. Working with people all day is really a strain for me. |
|  | 8. I feel burned out from my work. |
|  | 13. I feel frustrated by my job. |
|  | 14. I feel I’m working too hard on my job. |
|  | 16. Working with people directly puts too much stress on me. |
|  | 20. I feel like I’m at the end of my rope. |
| Depersonalization (DP) (5 items) | 1. I feel I treat some recipients as if they were impersonal objects. |
|  | 10. I’ve become more callous toward people since I took this job. |
|  | 11. I worry that this job is hardening me emotionally. |
|  | 15. I don’t really care what happens to some recipients. |
|  | 22. I feel recipients blame me for some of their problems. |
| Personal accomplishment (PA) (8 items) | 1. I can easily understand how my recipients feel about things. |
|  | 1. I deal very effectively with the problems of my recipients. |
|  | 9. I feel I’m positively influencing other people’s lives through my work. |
|  | 12. I feel very energetic. |
|  | 17. I can easily create a relaxed atmosphere with my recipients. |
|  | 18. I feel exhilarated after working closely with my recipients. |
|  | 19. I have accomplished many worthwhile things in this job. |
|  | 21. In my work, I deal with emotional problems very calmly. |
| BSRS-5 (5 items) |  |
|  | 1. Anxiety (feeling tense or high-strung) |
|  | 1. Depression (feeling depressed or in a low mood) |
|  | 1. Hostility (feeling easily annoyed or irritated) |
|  | 1. Inferiority (Interpersonal sensitivity, feeling inferior to others) |
|  | 1. Poor sleep quality (Additional symptoms, having trouble falling asleep) |

| Supplement table 2  A. Correlation between the items of BSRS-5 and domain of MBI | | | | | | | | | |
| --- | --- | --- | --- | --- | --- | --- | --- | --- | --- |
|  | | | | | | | | | |
| **Variables** | | **Maslach Burnout Inventory (MBI)** | | | | | | | |
|  |  | **Emotional exhaustion** | |  | **Depersonalization** | |  | **Personal accomplishment** | |
|  |  | **Pearson's correlation coefficient** | **p value** |  | **Pearson's correlation coefficient** | **p value** |  | **Pearson's correlation coefficient** | **p value** |
| BSRS-5 (5 item) | | |  |  |  |  |  |  |  |
|  | Anxiety | 0.56 | <0.001 |  | 0.56 | <0.001 |  | -0.03 | 0.09 |
|  | Depression | 0.61 | <0.001 |  | 0.55 | <0.001 |  | -0.09 | <0.001 |
|  | Hostility | 0.60 | <0.001 |  | 0.55 | <0.001 |  | -0.07 | 0.001 |
|  | Inferiority | 0.48 | <0.001 |  | 0.51 | <0.001 |  | -0.08 | <0.001 |
|  | Poor sleep quality | 0.47 | <0.001 |  | 0.40 | <0.001 |  | -0.06 | 0.002 |
| BSRS-5 score | | 0.65 | <0.001 |  | 0.61 | <0.001 |  | -0.08 | <0.001 |

B. Correlation between the items of BSRS-5 and domain of MBI stratified by age and gender

| **Variables** | | **Maslach Burnout Inventory (MBI)** | | | | | | | |
| --- | --- | --- | --- | --- | --- | --- | --- | --- | --- |
|  |  | **Emotional exhaustion** | |  | **Depersonalization** | |  | **Job control (Personal accomplishment)** | |
|  |  | **Pearson's correlation coefficient** | **p value** |  | **Pearson's correlation coefficient** | **p value** |  | **Pearson's correlation coefficient** | **p value** |
| **Men<30 years** | |  |  |  |  |  |  |  |  |
| BSRS-5 (5 item) | | |  |  |  |  |  |  |  |
|  | Anxiety | 0.62 | <0.001 |  | 0.57 | <0.001 |  | -0.15 | 0.20 |
|  | Depression | 0.61 | <0.001 |  | 0.60 | <0.001 |  | -0.18 | 0.13 |
|  | Hostility | 0.69 | <0.001 |  | 0.62 | <0.001 |  | -0.35 | 0.003 |
|  | Inferiority | 0.55 | <0.001 |  | 0.61 | <0.001 |  | -0.15 | 0.23 |
|  | Poor sleep quality | 0.52 | <0.001 |  | 0.48 | <0.001 |  | -0.19 | 0.12 |
| BSRS-5 score | | 0.69 | <0.001 |  | 0.67 | <0.001 |  | -0.23 | 0.06 |
|  |  |  |  |  |  |  |  |  |  |
| **Men≧30 years** | | |  |  |  |  |  |  |  |
| BSRS-5 (5 item) | | |  |  |  |  |  |  |  |
|  | Anxiety | 0.60 | <0.001 |  | 0.54 | <0.001 |  | -0.18 | 0.01 |
|  | Depression | 0.69 | <0.001 |  | 0.43 | <0.001 |  | -0.23 | 0.001 |
|  | Hostility | 0.64 | <0.001 |  | 0.41 | <0.001 |  | -0.14 | 0.04 |
|  | Inferiority | 0.57 | <0.001 |  | 0.50 | <0.001 |  | -0.28 | <0.001 |
|  | Poor sleep quality | 0.50 | <0.001 |  | 0.37 | <0.001 |  | -0.23 | <0.001 |
| BSRS-5 score | | 0.71 | <0.001 |  | 0.53 | <0.001 |  | -0.25 | <0.001 |
|  |  |  |  |  |  |  |  |  |  |
| **Women<30 years** | | |  |  |  |  |  |  |  |
| BSRS-5 (5 item) | | |  |  |  |  |  |  |  |
|  | Anxiety | 0.52 | <0.001 |  | 0.54 | <0.001 |  | -0.0003 | 0.99 |
|  | Depression | 0.62 | <0.001 |  | 0.57 | <0.001 |  | -0.06 | 0.08 |
|  | Hostility | 0.61 | <0.001 |  | 0.54 | <0.001 |  | -0.07 | 0.04 |
|  | Inferiority | 0.48 | <0.001 |  | 0.50 | <0.001 |  | -0.04 | 0.24 |
|  | Poor sleep quality | 0.47 | <0.001 |  | 0.43 | <0.001 |  | -0.04 | 0.20 |
| BSRS-5 score | | 0.64 | <0.001 |  | 0.61 | <0.001 |  | -0.05 | 0.13 |
|  |  |  |  |  |  |  |  |  |  |
| **Women≧30 years** | | |  |  |  |  |  |  |  |
| BSRS-5 (5 item) | | |  |  |  |  |  |  |  |
|  | Anxiety | 0.56 | <0.001 |  | 0.56 | <0.001 |  | -0.06 | 0.02 |
|  | Depression | 0.59 | <0.001 |  | 0.54 | <0.001 |  | -0.11 | <0.001 |
|  | Hostility | 0.59 | <0.001 |  | 0.56 | <0.001 |  | -0.06 | 0.02 |
|  | Inferiority | 0.46 | <0.001 |  | 0.50 | <0.001 |  | -0.09 | <0.001 |
|  | Poor sleep quality | 0.45 | <0.001 |  | 0.37 | <0.001 |  | -0.06 | 0.01 |
| BSRS-5 score | | 0.64 | <0.001 |  | 0.61 | <0.001 |  | -0.09 | <0.001 |
